# Detection of an endangered aquatic heteropteran using environmental DNA in a wetland ecosystem

**DOI:** 10.1098/rsos.170568

**Published:** 2017-07-19

**Authors:** Hideyuki Doi, Izumi Katano, Yusuke Sakata, Rio Souma, Toshihiro Kosuge, Mariko Nagano, Kousuke Ikeda, Koki Yano, Koji Tojo

**Affiliations:** 1 Graduate School of Simulation Studies, University of Hyogo, 7-1-28, Minatojima-minamimachi, Chuo-ku, Kobe 650-0047, Japan; 2 School of Human Science and Environment, University of Hyogo, 1-1-12 Shinzaike-Honcho, Himeji 670-0092, Japan; 3 Graduate School of Human Science and Environment, University of Hyogo, 1-1-12 Shinzaike-Honcho, Himeji 670-0092, Japan; 4 Faculty of Science, Nara Women's University, Kitauoyahigashi-machi, Nara 630-8506, Japan; 5 Pacific Consultants Co., Ltd, 3-22, Kanda-Nishikicho, Chiyoda-ku, Tokyo 101-8462, Japan; 6 Graduate School of Environmental Science, Hokkaido University, Kita 10 Nishi 5, Kita-ku, Sapporo, Hokkaido 060-0808, Japan; 7 Department of Biology, Faculty of Science, Shinshu University, 3-1-1 Asahi, Matsumoto 390-8621, Japan

**Keywords:** real-time PCR, species detection, eDNA, conservation, endangered species, heteroptera

## Abstract

The use of environmental DNA (eDNA) has recently been employed to evaluate the distribution of various aquatic macroorganisms. Although this technique has been applied to a broad range of taxa, from vertebrates to invertebrates, its application is limited for aquatic insects such as aquatic heteropterans*. Nepa hoffmanni* (Heteroptera: Nepidae) is a small (approx. 23 mm) aquatic heteropteran that inhabits wetlands, can be difficult to capture and is endangered in Japan. The molecular tool eDNA was used to evaluate the species distribution of *N. hoffmanni* in comparison to that determined using hand-capturing methods in two regions of Japan. The eDNA of *N. hoffmanni* was detected at nearly all sites (10 eDNA-detected sites out of 14 sites), including sites where *N. hoffmanni* was not captured by hand (five eDNA-detected sites out of six captured sites). Thus, this species-specific eDNA technique can be applied to detect small, sparsely distributed heteropterans in wetland ecosystems. In conclusion, eDNA could be a valuable technique for the detection of aquatic insects inhabiting wetland habitats, and could make a significant contribution to providing distribution data necessary to species conservation.

## 1. Introduction

Freshwater biodiversity, including that of aquatic invertebrates, is the overriding conservation priority of the International ‘Water for Life’ Decade for Action [1]. To investigate the biodiversity and distribution of aquatic organisms, environmental DNA (eDNA)—genetic material obtained directly from environmental samples that are collected without specifically targeting the organisms of interest—has recently been considered as a useful technique [2–4], including aquatic invertebrates [5–7]. This technique can be applied to the study of many species of macroorganisms inhabiting various freshwater habitats, including rivers [8–14], and lakes and ponds [15–19].

The eDNA would also be applied to estimate species abundance/biomass based on eDNA concentrations as determined by quantitative PCR and the detection rates of PCR replicates [20–24]. These studies have demonstrated positive relationships between abundance and/or biomass and eDNA concentrations/detection rates in the field [10,22,24], and support the use of eDNA techniques for estimating species biomass/abundance. However, there are larger variabilities in the estimations, especially in the field owing to the uncertainty of eDNA transports and releasing [23].

Aquatic and semi-aquatic insects of the suborder Heteroptera represent a significant component of the global aquatic insect population [25], and play an important role in food webs as predatory species in freshwater habitats (e.g. wetlands) [26]. In addition, aquatic heteropterans require specific habitats, making them vulnerable to the loss of physical integrity in aquatic systems, where heteropteran diversity often correlates with the physical integrity of the environment [27]. Thus, this feature of heteropteran distribution makes them valuable bioindicators [27]. However, to use aquatic heteropterans as bioindicators, it is first necessary to evaluate the distribution of aquatic heteropteran species both temporally and spatially. The eDNA techniques would be a potential tool for investigating aquatic heteropteran distributions. The technique has been applied to various taxa, from vertebrates to invertebrates such as crustaceans [5,9], amphibians [10,28] and fishes [8,14,19,20,24]. Although eDNA could be useful for the detection of aquatic insects, its application in this capacity may be limited [29] by the relatively smaller body size of aquatic insects (including heteropterans) than that of invertebrate and vertebrate species in previously published studies, especially for rare species. Although some previous studies performed eDNA surveys for aquatic insect species such as dragonfly [5] and chironomids [7], the application of the eDNA method to detect aquatic insects has still not been well tested.

Here, eDNA was used to evaluate the distribution of *Nepa hoffmanni* Esaki, 1925 (Nepidae, Heteroptera), an aquatic hemipteran that is endangered in Japan. The performance of this technique to detect *N*. *hoffmanni* was compared to that of the hand-capturing method. In addition, the correlation between eDNA detection rates and the abundance of species recorded by hand-capturing was investigated to evaluate the use of eDNA in estimating species abundance.

## 2. Material and methods

### 2.1. Study species

*Nepa hoffmanni* is an aquatic heteropteran that is distributed in the central part of Honshu island, Japan. *Nepa hoffmanni* is an endangered species found on the Red Lists of some Japanese Prefectures (e.g. critically endangered (CR) in Hyogo Prefecture, and near threatened (NT) in Aichi Prefecture, including Toyota City (Search System of Japanese Red Data Book, http://www.jpnrdb.com/index.html, accessed 22 December 2016)). In Kuwana City, Mie Prefecture, *N. hoffmanni* is listed as a natural monument species, which is a protected species by the government. This species is one of the smallest aquatic heteropterans in Japan, with an approximate body length of 23 mm, and is generally distributed in wetlands and small streams [30,31].

### 2.2. Field survey for environmental DNA sample and hand-capturing methods

**Figure 1.**
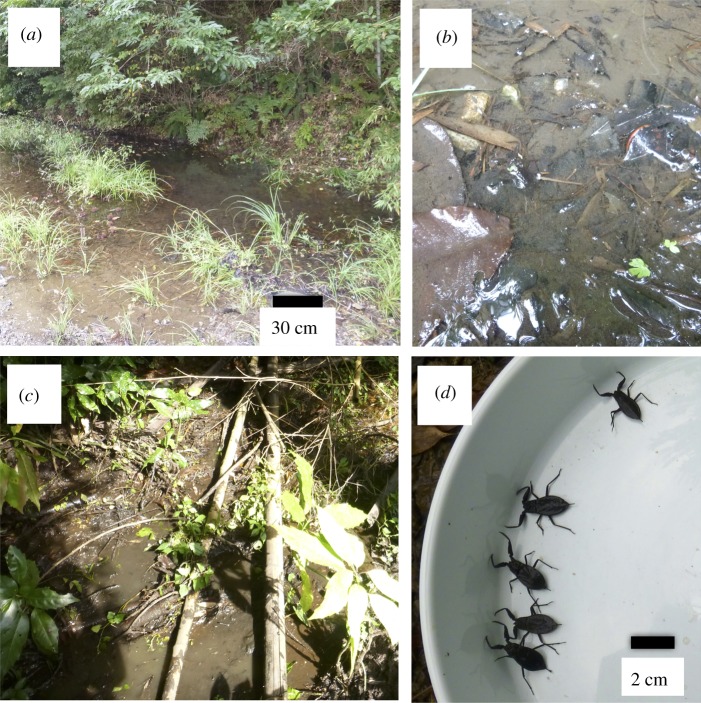
Photos of study site (site 11, *a*), a sampling habitat (site 11, *b*), study site (site 13, *c*) and *N. hoffmanni* collected at site 13 (*d*).

Surface water collections for eDNA and capturing surveys of *N. hoffmanni* were performed during the active season [30], from August to December 2014 and October 2016 at 14 swamp sites in Hyogo Prefecture and Toyota, Japan. The swamps had a small area, approximately, less than 10 × 10 m, namely less than 100 m^2^ (e.g. figure 1*a,c*). The locations of the sites were approximately 34°46′–34°51′ N, 135°19′–134°54′ E for Hyogo, and 35°03′–35°14′ N, 137°24′ E for Toyota (N.B.: only broad site coordinates are provided so as not to disclose the precise locations of the habitats). The two regions chosen for this study are known to be major habitats for *N. hoffmanni* [30]. After water sampling at each site (see the following section for more detail), the hand-capturing of *N. hoffmanni* was performed for a period of 20 min. In the swamp sites, a person (Y.S. or T.K.) visually observed the water surface from the shore and in the swamp, and captured the individuals *N. hoffmanni* by hand. During the 20 min survey, we could observe the whole area of small swamps (less than 100 m^2^) several times. The time for searching was referred by the previous studies, which collected aquatic heteropterans in wetlands, including swamps [32,33]. In these studies, they took 2–30 min for a much larger area (greater than 100 m^2^) for the survey.

### 2.3. Environmental DNA collection and extraction from water samples

Surface water (1 l) was collected just prior to hand-capturing at each site. Water samples were carefully collected to avoid sediment contamination in the water, as the water depth of the sampling sites was very low (0–10 cm) (figure 1). At site 12, water was collected from the stream outflow of the swamp owing to very low water depth. Prior to collection, collection bottles were bleached with 10% bleach and washed with DNA-free distilled water (ultrapure deionized filtration). Water samples were vacuum-filtered through 47 mm GF/F glass filters (pore size: approx. 0.7 µm, GE Healthcare, Little Chalfont, UK). Filters were wrapped in commercial aluminium foil and stored at –20°C until eDNA extraction. An ‘equipment blank’ (1 l DNA-free distilled water) and a ‘cooler blank’ were incorporated as negative controls for each filtering and sampling step, respectively. As a cooler blank, 1 l of ultrapure water was brought from the laboratory to the field sampling sites, where the cooler blank was treated identically to water-sampling bottles with the exception that it was not opened at the field sites [24]. In the laboratory, cooler and equipment blanks were filtered as negative controls after filtering the collected samples on each sampling day. These negative controls, tested along with the field samples, allowed for the identification of field preparation/transportation, filter equipment or background contamination in eDNA detection [24]. Each piece of equipment used in filtration was soaked in a 10% bleach solution for 5 min and rinsed by distilled water (DW) prior to use.

DNA was extracted from filters according to the methods of Uchii *et al*. [19], using the DNeasy Blood and Tissue Kit for DNA purification (Qiagen, Hilden, Germany) and Salivette tube (Sarstedt, Nümbrecht, Germany). First, filters were incubated by submersion in a mixed buffer (400 µl of buffer AL and 40 µl of Proteinase K; Qiagen) using a Salivette tube in a dry oven at 56°C for 30 min. Then, Salivette tubes with filters were centrifuged at 5000*g* for 5 min at 20°C, 220 µl of TE (Tris-EDTA) buffer (pH: 8.0; 10 mM Tris–HCl and 1 mM EDTA) was added to the filter, and tubes were centrifuged again at 5000*g* for 5 min. The DNA in the eluted solution was purified using a DNeasy Blood & Tissue Kit (Qiagen) following the manufacturer's protocol. The final volume of the extracted sample was 100 µl (with buffer AE from the DNeasy Blood & Tissue Kit) and samples were stored at –20°C for qPCR.

### 2.4. Primer and probe design for *Nepa hoffmanni*

**Figure 2.**
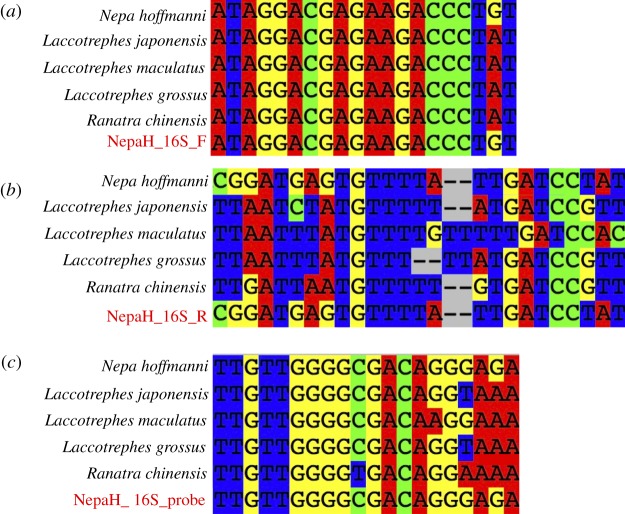
PCR primer/probe sets. Primers (*a*) NapaH_16S_F, (*b*) NapaH_16S_R and probe (*c*) NapaH_16S_probe were designed for *N. hoffmanni* based on 16S rRNA and consensus sequence information from *N. hoffmanni* and other Napidae species found Japan. Sequence data were from accession numbers: *N. hoffmanni*: LC213560, LC213561, LC213562, LC213563, LC213564, LC213565, LC213568, LC213571, LC213572 and LC213573; *L. japonensis*: LC213569, LC213570, LC213574 and LC213575; *L. maculatus*: LC213566; and *L. grossus*: LC213567. Note the sequence of NapaH_16S_R was a reverse complement of the sequence indicated in this figure.

To detect the DNA of *N. hoffmanni* using real-time PCR, species-specific primers were developed to amplify a fragment of the 16S ribosome gene from mitochondrial DNA (mtDNA). The 135 sets of the forward–reverse primers and TaqMan probe for 100–150 bp fragment of the 16S region of *N. hoffmanni* mtDNA were provided by Primer3Plus (http://www.bioinformatics.nl/primer3plus). From the primers-probe sets, we visually selected the design using the alignment of the targeted and related species with checking the mismatching of sequence, especially in the 5′ or 3′ edges of primers. The design of the primers and TaqMan probe to amplify a 117 bp fragment of the 16S mtDNA were as follows (figure 2):

NepaH_16S_F (5′-ATAGGACGAGAAGACCCTGT-3′)

NepaH_16S_R (5′-ATAGGATCAATAAAACACTCATCCG-3′)

NepaH_16S_probe (5′-FAM- TTGTTGGGGCGACAGGGAGA-TAMRA-3′).

The specificities of the primers and probe were checked *in silico* with homologous sequences from other Nepinae species inhabiting Japan from the National Center for Biotechnology Information (NCBI, http://www.ncbi.nlm.nih.gov/) (*N. hoffmanni* accession numbers: LC213560, LC213561, LC213562, LC213563, LC213564, LC213565, LC213568, LC213571, LC213572 and LC213573; *Laccotrephes japonensis* accession numbers: LC213569, LC213570, LC213574 and LC213575; *Laccotrephes maculatus* accession number: LC213566; and *Laccotrephes grossus* accession number: LC213567 were obtained from unpublished sequences generated in our laboratory). Species from Japan of the order Nepinae were not detected during the *in silico* screen for specificity, which was performed using Primer-BLAST (http://www.ncbi.nlm.nih.gov/tools/primer-blast/).

### 2.5. Environmental DNA detection by real-time PCR

eDNA was quantified by real-time PCR using the PikoReal Real-Time PCR System (Thermo Scientific, Waltham, MA, USA). Each TaqMan reaction (10 µl total volume) contained 900 nM of each primer (NapaH_16S_F and _R), 125 nM TaqMan probe (NapaH_16S_probe), 5 µl real-time PCR master mix (TaqMan Environmental Master Mix 2.0; Thermo Scientific), 0.2 µl AmpErase^®^ Uracil *N*-Glycosylase (Thermo Scientific), 2 µl of the DNA solution and DNA-free water. To avoid contamination, the aforementioned PCR set-up and procedure were performed in two separate rooms. The PCR (eight replicates) was performed as follows: 2 min at 50°C, 10 min at 95°C and 55 cycles of 15 s at 95°C and 60 s at 60°C. The non-template control was performed in eight replicates per PCR. The results of the PCR were analysed using PikoReal software v. 2.2.248.601 (Thermo Scientific). All of the aforementioned real-time PCR procedures were performed according to the MIQE [34].

To confirm primer specificity, an *in vivo* test for the primer/probe set (NepaH_16S_F, R, probe) was also performed using the extracted DNA (100 pg per PCR reaction, *n* = 8) for all Nepinae species found in Japan (*L*. *japonensis*, *L. maculatus* and *L. grossus*). Also, qPCR amplicons were sequenced directly from a positive PCR from each site (*n* = 9) after treatment with ExoSAP-IT (USB Corporation, Cleveland, OH, USA). Sequences were determined by a commercial sequencing service (Eurofins Genomics, Tokyo, Japan).

### 2.6. PCR inhibition test

As a measure of the relative degree of PCR inhibition, the Ct shift was compared between the samples and controls with the same number of known target DNA copies [35,36]. The Ct is defined as the number of cycles required for enough amplified PCR product to accumulate that it surpasses a threshold recognized by the real-time PCR instrumentation. Ct is inversely related to starting quantity of target DNA in a reaction and is used to calculate this quantity. The presence of PCR inhibitors will shift (delay) the Ct for a given quantity of template DNA. To test for inhibition in the DNA samples, 1 µl of the plasmid including the cytochrome *b* gene from *Trachurus japonicus* (1.5 × 10^2^ copies), a marine fish and does not inhabit the streams sampled, was added to the PCR temperate with decreasing 1 µl of DNA-free DW. The primer and probe set used was that reported by Yamamoto *et al*. [37]: forward primer: 5′-CAGATATCGCAACCGCCTTT-3′; reverse primer: 5′-CCGATGTGAAGGTAAATGCAAA-3′; probe: 5′-FAM-TATGCACGCCAACGGCGCCT-TAMRA-3′. The presence of PCR inhibitors was evaluated ΔCt (Ct_positive control _– Ct_sample_). ΔCt ≥ 3 cycles was considered to be evidence of inhibition [35].

### 2.7. Statistical analyses

Cohen's *κ* test [38] was used to compare the detection between the hand-capturing method and eDNA. Cohen's *κ* value was calculated by comparing the number of sites in which *N. hoffmanni* were detected by the hand-capturing method and eDNA detection in the sampled water by real-time PCR. Cohen's *κ* should be between 0 and |1|, with |1| referring to the highest matching for the proportion [38]. Cohen's *κ* value for the proportion was |1|, indicating that the proportions were perfectly matched [38]. The null hypothesis of Cohen's *κ* test was that Cohen's *κ* value for the proportion differed from 0, indicating that the proportions were not matched. To compare the number of individuals per 20 min hand-capturing and eDNA detection, Spearman's rank correlation was calculated, as the values analysed were counted numbers, and the significance was tested. We performed Spearman's rank correlation test for all sites and the sites with the presence of the species. The *α* for all statistical testing was set at 0.05. All statistical analyses and graphics were conducted in R v. 3.3.2 [39] with ‘irr’ v. 0.84 for Cohen's *κ* test, ‘cor.test’ for Spearman's rank correlation and ‘ggplot2’ v. 2.1.0 for graphics.

## 3. Results

### 3.1. Testing species-specificity of PCR primers/probe

The extracted DNA of *N. hoffmanni* was detected using the primer-probe set by real-time PCR, while that of the other tested species (*L. japonensis*, *L. maculatus* and *L. grossus*) was not. The direct sequencing of the PCR amplicons confirmed that the PCR amplicons detected in this study were from *N. hoffmanni.*

### 3.2. Detection of species distribution by environmental DNA and hand-capturing

**Figure 3.**
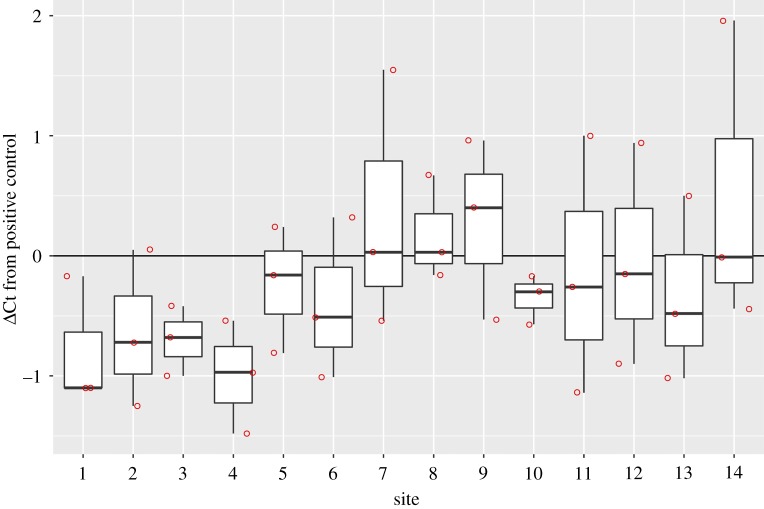
ΔCt values from internal positive controls via PCR inhibition test for samples collected from sites 1–14. The bold line in the box indicates the median value and upper and lower limits of the box. Whisker plots indicate the first and third quartiles and ±1.5 × interquartile range, respectively. The red dots represent each data point.

The eDNA of *N. hoffmanni* was detected at 10 sites, including sites where individuals were captured by hand (table 1). The eDNA of *N. hoffmanni* was detected at nearly all sites (10 eDNA-detected sites out of 14 sites), including sites where *N. hoffmanni* was not captured by hand (five eDNA-detected sites out of six captured sites). The result of Cohen's *κ* test was not significant with low *κ* value (*κ* = 0.192, *z* = 0.854, *p* = 0.393, *n* = 14), indicating that the proportions of the sites in which *N. hoffmanni* were detected between the hand-capturing method and eDNA detection were not significantly matched. In other words, eDNA was detected at four sites where *N. hoffmanni* was not captured, and was not detected at a site where *N. hoffmanni* was captured (site 11, table 1). The ΔCt values from the internal controls of all samples were lower than 3 (figure 3), indicating that they were lower than the inhibition criteria according to Hartman *et al*. [35]. PCR inhibition was not significant for all samples according to the qPCR estimation using Environmental Master Mix 2.0 (Thermo Scientific).

**Table 1.** Site locations, observed number of hand captured *N. hoffmanni* (per 20 min) and instances of positive eDNA detection per eight PCR replicates.

site	region	altitude (m)	sampling date	observed individuals	eDNA	remarks
1	Hyogo Pref.	190	12 Aug 2014	0	1/8	the species was previously observed^a^
2	Hyogo Pref.	187	12 Aug 2014	0	1/8	the species was observed in the upper pond during our survey
3	Hyogo Pref.	188	17 Sep 2014	0	0/8	
4	Hyogo Pref.	80	13 Aug 2014	0	0/8	
5	Hyogo Pref.	79	16 Sep 2014	0	1/8	
6	Hyogo Pref.	100	6 Dec 2014	1	4/8	
7	Hyogo Pref.	100	6 Dec 2014	0	3/8	
8	Hyogo Pref.	95	6 Dec 2014	1	1/8	
9	Hyogo Pref.	63	6 Dec 2014	3	5/8	
10	Toyota	228	10 Oct 2016	8	8/8	
11	Toyota	245	10 Oct 2016	1	0/8	low water depth (0–1 cm)
12	Toyota	240	10 Oct 2016	0	0/8	low water depth (0–1 cm)
13	Toyota	120	10 Oct 2016	8	1/8	the habitat was segmented by terrestrial plants
14	Toyota	114	10 Oct 2016	0	1/8	

^a^Harima Wetland Research 2013, personal observation.

**Figure 4.**
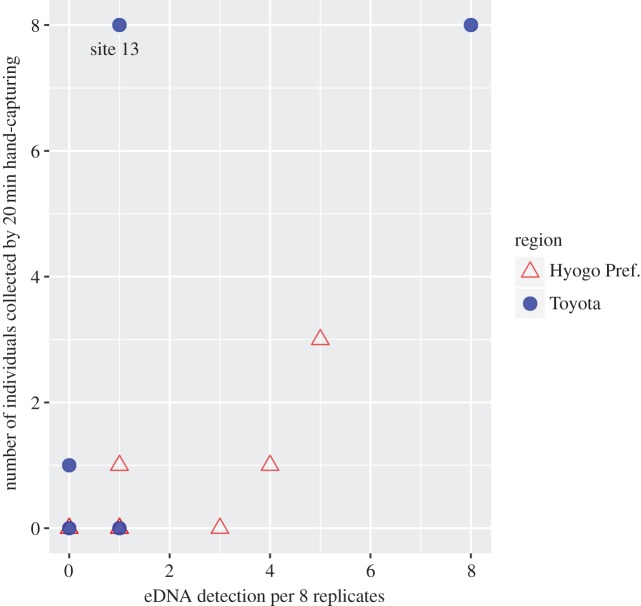
Biplot between eDNA detection per eight PCR replicates and number of individuals collected in the study area during a 20 min period. The correlation was not significant (*ρ* = 0.50, *p* = 0.068, *n* = 14).

There was no significant correlation between eDNA detection in eight PCR replicates and the number of individuals collected in the study areas (figure 4, Spearman's rank correlation, *ρ* = 0.50, *p* = 0.068, *n* = 14, *ρ* = 0.51, *p* = 0.051, *n* = 5 with presence sites). Excluding the data from site 13 which has largely segmented habitat by terrestrial plants (figure 1*c* and table 1), the relationships between the eDNA detection rate and the collected number of the species were statistically significant (*ρ* = 0.57, *p* = 0.044, *n* = 13).

## 4. Discussion

We performed this study to examine whether eDNA can be used as a means of species detection in a heteropteran insect. The results of this study confirm that the eDNA of *N. hoffmanni* can be detected at most study sites where species were collected directly, as well as from sites where this species has been previously recorded. The habitats in question are muddy in nature, with a large quantity of decomposed leaf matter (figure 1), which probably increases the content of humic acid, a recognized PCR inhibitor [40]. However, we did not find any sign of PCR inhibition in the samples analysed using Environmental Master Mix 2.0, which is suitable for environmental samples containing PCR inhibitors such as humic acid. The eDNA detected from water samples is known to be related to the abundance/biomass of the species [20–22,24]. Even though *N. hoffmanni* has relatively low abundance (a few individuals per square metre) and biomass (the lower abundance × smaller body mass; less than 23 mm, body length) at the study sites, eDNA can be detected using the 1 l water-sampling method described in this study. The water volume was larger than that by Thomsen *et al*. [5] to detect aquatic insects (i.e. 15 ml for eDNA); however, the larger water volume may allow the detection of the eDNA signals from such small insect species. Also, in this study, we just collected a single sample from each swamp site, although the heterogeneity of eDNA distribution has been suggested [41–43]. Further studies need to increase the detectability with increasing the sample size of water collection and sampling strategy (e.g. water volume) from the site. From the result, we should note the possibility of false-positive detection by real-time PCR, especially lower positives in eight replicates (e.g. sites 1, 2, 5 and 14 without species observation and low detection rate (1/8) in table 1). Recently, a site occupancy-detection modelling framework was applied for eDNA study to evaluate the error rate for false positive/negatives in eDNA detection [44,45]. Such an approach may allow us to consider the detectability of eDNA with considering false positive/negative rates.

In addition, the results of eDNA and hand-capturing did not correspond to one another at all sites. In other words, eDNA was detected at sites where individuals were not directly captured, and eDNA was not detected at all sites where individuals were observed in the 20 min period (i.e. site 11), a phenomenon that has also been observed in previous studies [5,41]. Water depth at site 11 was very low, and thus water mixing in the swamp may be very limited. The limitation of water movement at the site may have resulted in a false negative in eDNA detection. These results indicate that the eDNA method may have a similar sensitivity for the detection of *N. hoffmanni* to that of the hand-capturing survey, and that the eDNA method can be tailored to specifically detect the distribution of *N. hoffmanni*. Thus, eDNA may be useful as a new monitoring tool for small and sparsely distributed aquatic insects in wetlands and other aquatic habitats. False negatives in eDNA should be carefully considered in the estimation of species distribution as the previous model suggested [45].

There were no significant statistical relationships between the eDNA detection rate and the hand-collected number of species. Moreover, eight positives were detected from eight replicates at the most abundant site (site 10; eight individuals were found), while only one positive was detected in the most abundant site (site 13). The removal of site 13 as an outlier resulted in a significant relationship between eDNA and hand-capturing methods, and thus the relationship was evaluated using data from site 14. Further evaluation is necessary to elucidate this relationship with larger sample size and using quantitative PCR with standard curve.

It is important to note that directly sampling for endangered species (e.g. *N. hoffmanni*) could have negative impacts on populations as well as ecosystems, especially in small wetlands or ecosystems limited in size. The use of eDNA techniques could be useful to mitigate these potential negative effects during monitoring as this tool allows for the non-invasive survey of endangered organisms.

In conclusion, the use of a real-time PCR primer/probe set, designed from the 16S rRNA region of *N. hoffmanni* mtDNA, resulted in the successful prediction of the distribution of *N. hoffmanni* using the eDNA method in comparison to that determined using the hand-capturing method. To the best of our knowledge, this is the first application of the eDNA technique to detect an aquatic heteropteran species, and may therefore be a valuable tool in the detection of small and/or sparsely populated aquatic insects. However, this study was only an initial step in the application of eDNA in the study of aquatic insects, and provided limited data to evaluate the temporal and spatial variations of eDNA detection. Thus, further study is required to estimate habitat-specific and seasonal variations.

## Data accessibility

Our data are deposited at the Dryad Digital Repository: http://dx.doi.org/10.5061/dryad.4rq73 [46].
